# Primary Headache Disorders at a Tertiary Health Facility in Lagos, Nigeria: Prevalence and Consultation Patterns

**DOI:** 10.1155/2014/782915

**Published:** 2014-01-23

**Authors:** Olajumoke Oshinaike, Oluwadamilola Ojo, Njideka Okubadejo, Olaitan Ojelabi, Akinola Dada

**Affiliations:** ^1^Department of Medicine, Lagos State University College of Medicine, GRA, Ikeja, Lagos, Nigeria; ^2^College of Medicine, University of Lagos, Idi-Araba, Lagos, Nigeria

## Abstract

*Background*. Primary headaches are underdiagnosed and undertreated, with a significant impact on social activities and work. *Aim.* To determine the last-year prevalence and health care utilization pattern of primary headaches at a tertiary centre. *Methods.* A cross-sectional study was carried out amongst staff of the Lagos State University Teaching Hospital in Lagos, Nigeria. 402 staff members were selected by simple random sampling and administered a detailed structured headache assessment questionnaire. Migraine and tension-type headache were diagnosed according to the criteria of the International Headache Society (2004). *Results.* The participants comprised 168 males and 234 females. The mean age was 36.9 ± 7.9 years. The overall headache prevalence was 39.3% with female predominance (*P* < 0.0001). Tension-type headache was the most prevalent at 72.8% and migraine at 18.9%. Unclassifiable headache constituted 8.2%. Migraine headache showed female preponderance (*P* = 0.000). 80.4% of participants did not seek medical consultation compared with 19.6% who did (*P* = 0.000). Of the latter, 83.9% consulted the general practitioner (GP), whilst 16.1% consulted the neurologist. *Conclusions.* Primary headache prevalence is high in our population. It is not recognised as that requiring care by most of the staff of this tertiary health facility; thus education is required to increase health care utilization.

## 1. Introduction

Headache is one of the most common neurological disorders [1] and accounts for multiple visits to the general physician and neurologist. Primary headaches cause significant disability with reduced efficiency, quality of life, and lost workdays [2–6]. Few receive appropriate diagnosis and adequate care. Migraine and tension-type headache are the most prevalent primary headache disorders.

Worldwide, the current global prevalence of primary headache is 47%; migraine headache, 10%; tension-type headache, 38%; and chronic daily headache, 3% [4]. The lifetime prevalence rates are higher: in men, 93% for headache of any kind, 8% for migraine, and 69% for tension-type headache. In women, lifetime prevalence is 99% for headache of any kind, 25% for migraine, and 88% for tension-type headache [7]. In Africa, data on headache prevalence is sparse. The 1-year prevalence of headache documented in rural south Tanzania was 23.1% [8], whilst in Ethiopia the 1-year prevalence of migraine was 3% (4.2% females and 1.7% males) with a peak age specific rate in the fourth decade [9]. Osuntokun et al. [10] in Nigeria documented the crude prevalence ratio of migraine headache to be 5.3 per 100 (5 per 100 in males and 5.6 per 100 in females).

The burden of headache is enormous. According to the World Health Organization (WHO), migraine ranks 19th amongst all causes of years lived with disability (YLDs). Rasmussen et al. documented that the burden of tension-type headache is greater than that of migraine regarding absenteeism from work. In Sweden, migraine was noted to affect family, love life, and sex life almost as much as it affected work [11, 12]. Other studies have also documented significant effects on the partners of patients with migraine with 24% missing days of family or social activities and 12% avoiding plans for family and social activities due to proband's migraine. Some patients with migraine have reported the influence of the disease on their ability of good parenting, and a minority confirmed that they avoided having children because of their migraine [13, 14].

Headache burden can be reduced significantly by increasing awareness in the population, timely hospital visits, recognition of precipitating factors, and treatment. Published studies assessing the epidemiology of primary headache disorders are sparse in Nigeria. Knowledge of the prevalence and disability burden in this region would add to the framework of the Global campaign to reduce headache burden world-wide. We aimed to determine primary headache prevalence, its symptom profile, and pattern of health care utilization at a tertiary institution in urban Lagos, Nigeria, using the operational diagnostic criteria of the International Headache Society (IHS) [15].

## 2. Methodology

### 2.1. Study Site

The Lagos State University Teaching Hospital is a state owned tertiary institution located in Ikeja, a suburb of the city of Lagos and the capital of Lagos State, Nigeria. It provides easily accessible health care services for its staff and the people of Lagos state with a population of over 21 million.

### 2.2. Study Subjects

The study population included 402 workers of the Lagos State University Teaching Hospital, Lagos, Nigeria. The participants were selected from nurses, doctors, pharmacists, drivers, engineering staff, administrative staff, and security officers. The inclusion criterion was age 18 and above and being a staff of the hospital, whilst the exclusion criterion was refusal to participate in the study. Approval was granted from the Research and Ethics Committee of the Lagos state teaching hospital and consent was gotten from all participants.

### 2.3. Study Design

This was a hospital-based, cross-sectional survey in which the study participants were selected by systematic random sampling. The number of subjects from each department was determined by proportionate allocation based on the departmental staff strength.

### 2.4. Examination

The headache survey was performed by means of an interview based on a detailed pretested structured assessment questionnaire. The interviews were conducted under the supervision of the first author (O.O.Os). The headache assessment questionnaire contained an initial section for demographic data and the next section included a description of the current features of headache as well as its characteristics. Details of the research were communicated to the consenting participants at the beginning of the exercise. The participants were given the questionnaires to fill out and were recollected for review the following day. Subsequently, telephone interviews were conducted on all participants to corroborate the diagnosis reached based on the review of the questionnaires. Medical consultation was assessed by self-reported visits to the health care provider in the preceding year and treatment pattern was documented.

### 2.5. Diagnostic Criteria

Headache was diagnosed according to the criteria of the International Headache Society (2004) [15].

Migraine was diagnosed in subjects with recurrent, moderate to severe unilateral throbbing headache associated with nausea or vomiting or visual disturbances. The subjects with migraine were not subclassified. Tension-type headache was diagnosed when subjects suffered from bilateral or vertex tightness or pressure-like feeling in the absence of gastrointestinal or visual discomfort. Details of the diagnostic criteria for both migraine and tension-type headaches are shown in Appendix.

### 2.6. Data Analysis

All data were coded using the Statistical Package for the Social Sciences (SPSS) version 19.0.

The data were summarized using frequency tables, means, and standard deviations for continuous variables. Frequency and contingency tables were used for the categorical data. The level of significance was considered as *P* < 0.05.

## 3. Results

### 3.1. Demographic Characteristics of Study Population

A total of 500 questionnaires were distributed, of which 402 were returned, giving a participation rate of 80.4%. The remaining (19.6%) had either misplaced the questionnaire, did not wish to continue with the study, or were not available for telephone interview after completion of the questionnaires The 402 staff members that completed the questionnaires and had telephone interviews comprised 221 (55%) medical staff and 181 (45%) nonmedical staff. There were 168 (41.8%) males and 234 (58.2%) females. Their ages ranged between 20 and 55 years with a mean of 36.9 ± 7.9 years (median 38.8, range 35). The ages of the males ranged between 20 and 55 years with a mean of 35.9 ± 7.5 years (median 35.9, range 35), whilst that of the females ranged between 20 and 55 years with a mean of 37.6 ± 8.5 years (median 39, range 35).

### 3.2. Prevalence of Headache Types

**Figure 1:**
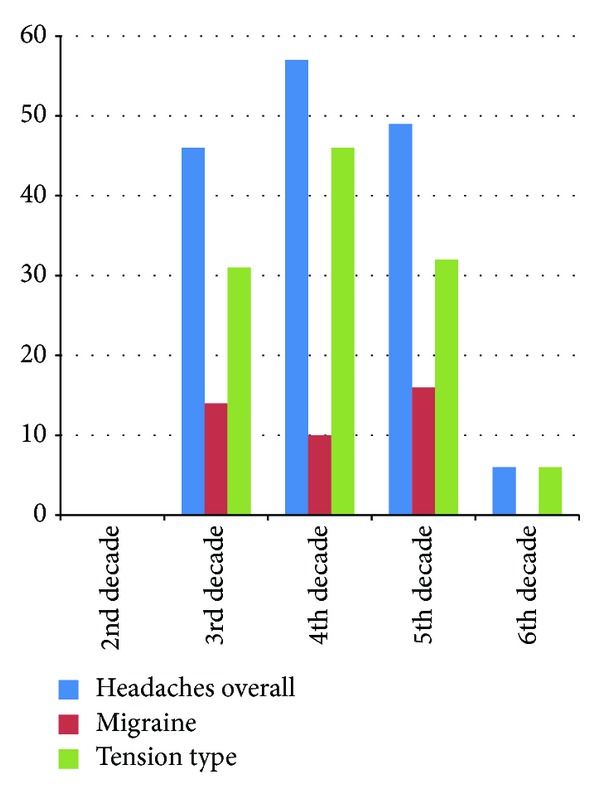
Prevalence of headache types in relation to age in decades.

**Figure 2:**
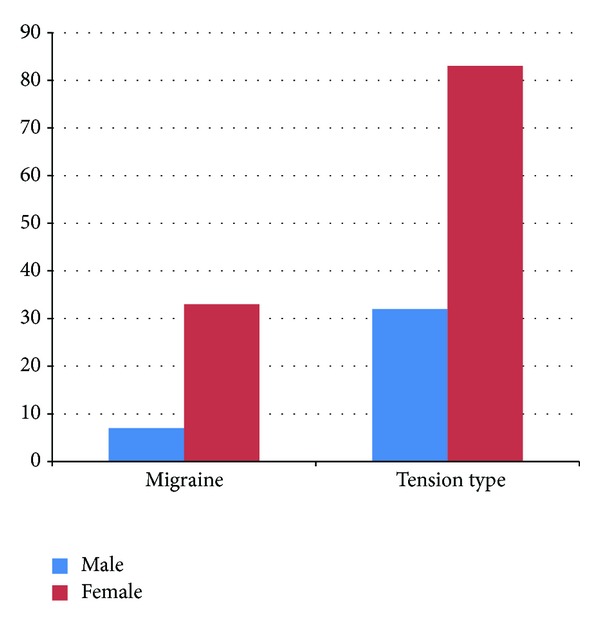
Gender difference in headache types.

Overall, a total of 158/402 (39.3%) staff members had recurrent headaches. The prevalence in males was 23.8% (40/168) and females 50.4% (118/234). Headache was significantly more common in females than in males. *P* < 0.001 (Figure 1) and in the 4th decade of life (Figure 2). The headache prevalence in medical staff staff members was 35.3% (78/221) compared to 42.5% (77/181) in nonmedical staff *P* = 0.069.

The prevalence of migraine was 18.9% (30/158) and it was significantly higher in females 28/118 (23.7%) than in males 2/40 (5%), *P* < 0.001. The mean age of onset of migraine headache was 19.2 ± 8.24 years. Physical activity was the main aggravating factor and occurred in 25% (10/40) of cases, whilst the relieving factors were rest in 62.5% (25/40) and over-the-counter analgesics in 17.5% (7/40) of cases.

The prevalence of tension-type headache was 72.8% (115/158). 83/115 (70.3%) of females had tension-type headache compared with 32/40 (80%) in males (*P* < 0.001). The mean age of onset of tension-type headache was 27.6 + 10.7 years. The most common aggravating factor was physical activity, occurring in 42.6% (49/115) of cases, whilst the relieving factors were rest in 57.4% (66/115) and over-the-counter analgesic use in 31.3% (36/115) of cases.

13/158 (8.2%) participants (7 females and 6 males) did not fulfill all criteria for migraine and tension-type headache and were classified as unclassifiable headache. No case of cluster headache was documented.

### 3.3. Pattern of Health Care Resource Utilization and Treatment

Participants who did not visit the health care provider for their headaches made up 127/158 (80.4%) compared with 31/158 (19.6%) who did (*P* < 0.001). Of the latter, 26/31 (83.9%) consulted the general practitioner (GP) whilst 5/31 (16.1%) the neurologist. Females made up 24/31 (77.4%) of those who sought medical consultation, whilst the males made up 7/31 (22.6%); *P* = 0.69. More participants with tension-type headache 21/31 (67.7%) sought medical consultation compared with those with migraine headache 5/31 (16.1%).

The overall treatment included simple analgesics, 5/31 (16.1%) nonsteroidal anti-inflammatory drugs (NSAIDS), 6/31 (19.4%) ergotamine derivatives, 10/31 (32.3%) amytriptilline, 9/31 (29%), and beta blockers 1/31 (3.2%). None had a prescription for triptans.

## 4. Discussion

This cross-sectional prevalent study in our urban population estimated the 1-year prevalence of primary headaches to be 39.3%. This finding is similar to the current global prevalence of 47% [4] and the 40% documented in the urban population of Brazil [16]. Ojini et al. [17] had earlier reported 46% at another teaching hospital in the same recruitment area as our centre of study. It is however lower than the population based study in Florianopolis, Brazil, which was 80.8% [18]. In comparison with studies conducted in working populations, Quesada-Vázquez and Rodríguez-Santanain Zimbabwe [19] had reported an overall headache prevalence of 37.1% which is also similar to our finding of 39.3%. Takele et al. in a population of textile mill workers however reported a much lower prevalence of 16.4% [20]. The differences in rates may be due to variation in the criteria for the definition of headache disorders and the differing age groups of the population studied.

More women compared with men had higher prevalent rates for primary headache in this study as has been previously reported [21, 22]. This has been attributed to the effect of female sex hormones specifically oestrogen.

We documented a prevalent rate of 18.9% for migraine in our study. This is higher than the global rate of 11% [7]. Takele et al. had reported a lower rate of 6.2%, whilst Quesada-Vázquez and Rodríguez-Santana reported a higher rate of 30.8%. One meta-analysis had indicated that the prevalence of migraine headache varied between different geographical regions, being lower in Europe than in North America but higher than in Asia and Africa [23]. Diversity of the population studied and racial differences in genetic vulnerability to migraine may also be contributory [24].

The well-known female preponderance in patients with migraine was also evident in our study. We found a significantly higher proportion of women with migraine headache, 8.2% compared to men, and 1.7%. The higher rates in women are thought to be due to factors such as sensitivity to the oestrogen hormone, genetics, and differences in response to stress and pain perception. Premenstrual migraines are known to occur during or after the time when the female hormones, oestrogen and progesterone, decrease to their lowest levels [25]. We noted that the prevalent rate of migraine increased with age until the 4th decade when it started to decline. Tekle Haimanot [9] in Ethiopia had also documented a decline after a peak in the fourth decade of life.

The prevalent rate of tension-type headache in our study was 72%. This finding is much higher than the 47.7% documented in Zimbabwe [19], the 25.5% by Quesada-Vázquez et al. in Cuba [26], and 11.2% reported in Oman [27]. It is however similar to the 78% and 86% reported by Rasmussen and Russell, respectively [28, 29]. There has been wide variations and differences in the epidemiology of tension-type headache across different cultures [4]. These variations may result from differences in study design, study population, inclusion or exclusion of cases of infrequent episodic TTH, and overlap with probable migraine, cultural and environmental differences, or even genetic factors [30].

A significant proportion, 80.4%, of the participants in our study did not visit the health care provider for treatment of their headache disorders even in the setting of a tertiary health care facility. This trend had been documented in previous studies. Lipton et al. reported that 34% of the participants in their study did not seek medical consultation for their headaches [31]. In another study, he also reported that almost half of the study population had not utilized the health care system for their headaches [32]. Majority of headache sufferers never seek health care utilization probably as a result of ignorance. In our study, a larger percentage 83.9% of those that utilized the health care system consulted the general practitioner (GP), whilst only 16.1% consulted the neurologist. Kristoffersen et al. [33] in their study reported 80% rate of GP consultation and 19% rate of neurology consultation which is similar to our findings.

## 5. Conclusion

Education on headache disorders as a substantial health problem and the benefit of health care utilization on the quality of life of headache sufferers need to be improved at all levels. Collaboration with policy-makers to plan and set up headache-related health-care services appropriate to local needs should be encouraged. Provision of additional training for general practitioners who attend to the bulk of patients with primary headache in this environment also cannot be over-emphasized.

## 6. Limitations

Our limitation includes lack of data on headache burden as this may probably explain the low percentage of health care utilization.

## Appendix

The International Classification of Headache Disorders (2004).

*Migraine Headache*

- ≥5 attacks lasting 4–72 hours
- ≥2 of the following 4
  - Unilateral location
  - Pulsating quality
  - Moderate or severe intensity
  - Aggravation by routine physical activity
- ≥1 of the following
  - Nausea and/or vomiting
  - Photophobia and phonophobia
- Not attributable to any other disorder

*Tension-Type Headache*

- ≥10 attacks lasting 30 minutes to 7 days
- ≥2 of the following 4
  - Bilateral location
  - Pressing/tightening (non-pulsating) quality
  - Mild or moderate intensity
  - Not aggravated by routine physical activity
- No nausea or vomiting
- One or either photophobia or phonophobia
- Not attributed to another disorder.
